# Monocyte distribution width (MDW) performance as an early sepsis indicator in the emergency department: comparison with CRP and procalcitonin in a multicenter international European prospective study

**DOI:** 10.1186/s13054-021-03622-5

**Published:** 2021-06-30

**Authors:** Pierre Hausfater, Neus Robert Boter, Cristian Morales Indiano, Marta Cancella de Abreu, Adria Mendoza Marin, Julie Pernet, Dolores Quesada, Iris Castro, Diana Careaga, Michel Arock, Liliana Tejidor, Laetitia Velly

**Affiliations:** 1 Emergency Department, Hôpital Pitié-Salpêtrière, APHP-Sorbonne Université, 83 Boulevard de l’hôpital, 75651 Paris Cedex 13, France; 2 Sorbonne Université, GRC-14 BIOSFAST, Paris, France; 3 UMR INSERM 1166, IHU ICAN, Sorbonne Université, Paris, France; 4 Emergency Department, Hospital Universitari Germans Trias I Pujol, Badalona, Spain; 5 Universitat Autònoma de Barcelona, Badalona, Spain; 6 Biochemisty and Emergency Biology Department, Hôpital Pitié-Salpêtrière, APHP-Sorbonne Université, Paris, France; 7 Laboratory Medicine Department, Laboratori Clinic Metropolitana Nord, Hospital Universitari Germans Trias I Pujol, Badalona, Spain; 8 Microbiology Department, Laboratori Clinic Metropolitana Nord, Hospital Universitari Germans Trias I Pujol, Badalona, Spain; 9 Beckman Coulter, Inc., Miami, FL USA

**Keywords:** Monocyte volume distribution width, MDW, Procalcitonin, C-reactive protein, Emergency department, Sepsis

## Abstract

**Background:**

Early sepsis diagnosis has emerged as one of the main challenges in the emergency room. Measurement of sepsis biomarkers is largely used in current practice to improve the diagnosis accuracy. Monocyte distribution width (MDW) is a recent new sepsis biomarker, available as part of the complete blood count with differential. The objective was to evaluate the performance of MDW for the detection of sepsis in the emergency department (ED) and to compare to procalcitonin (PCT) and C-reactive protein (CRP).

**Methods:**

Subjects whose initial evaluation included a complete blood count were enrolled consecutively in 2 EDs in France and Spain and categorized per Sepsis-2 and Sepsis-3 criteria. The performance of MDW for sepsis detection was compared to that of procalcitonin (PCT) and C-reactive protein (CRP).

**Results:**

A total of 1,517 patients were analyzed: 837 men and 680 women, mean age 61 ± 19 years, 260 (17.1%) categorized as Sepsis-2 and 144 patients (9.5%) as Sepsis-3. The AUCs [95% confidence interval] for the diagnosis of Sepsis-2 were 0.81 [0.78–0.84] and 0.86 [0.84–0.88] for MDW and MDW combined with WBC, respectively. For Sepsis-3, MDW performance was 0.82 [0.79–0.85]. The performance of MDW combined with WBC for Sepsis-2 in a subgroup of patients with low sepsis pretest probability was 0.90 [0.84–0.95]. The AUC for sepsis detection using MDW combined with WBC was similar to CRP alone (0.85 [0.83–0.87]) and exceeded that of PCT. Combining the biomarkers did not improve the AUC. Compared to normal MDW, abnormal MDW increased the odds of Sepsis-2 by factor of 5.5 [4.2–7.1, 95% CI] and Sepsis-3 by 7.6 [5.1–11.3, 95% CI].

**Conclusions:**

MDW in combination with WBC has the diagnostic accuracy to detect sepsis, particularly when assessed in patients with lower pretest sepsis probability. We suggest the use of MDW as a systematic screening test, used together with qSOFA score to improve the accuracy of sepsis diagnosis in the emergency department.

*Trial Registration* ClinicalTrials.gov (NCT03588325).

**Supplementary Information:**

The online version contains supplementary material available at 10.1186/s13054-021-03622-5.

## Introduction

A significant proportion of patients developing sepsis enter the health system through an emergency department (ED) [1–4]. Because the surviving sepsis campaign promotes targeting short-term specific, goal-directed therapy bundles, early sepsis diagnosis has therefore emerged as one of the main challenges for emergency physicians [5]. Although the systemic inflammatory response syndrome (SIRS) or quick Sequential Organ Failure Assessment (qSOFA) score are helpful clinical tools for sepsis suspicion during early triage, sepsis diagnosis may still be delayed or misdiagnosed [4, 6]. So, measurement of sepsis biomarkers is largely used in current practice to improve the diagnosis accuracy [7].

MDW, a hematologic parameter measured as part of the complete blood count with differential (CBC-DIFF) and describing the size distribution of circulating monocytes, was shown in two recent North American studies [8–10] to be a valuable new sepsis biomarker for the early detection of patients in the emergency department. However, little is known about the performance of MDW and how it compares to those of the most frequently used sepsis biomarkers: C-reactive protein (CRP) and procalcitonin (PCT) [11].

The primary objective of this study was to confirm the clinical validity and performance of MDW to identify patients who developed sepsis in a European ED population. Secondary objectives were to compare the performance of MDW with CRP and PCT and to verify MDW cutoff for tripotassium ethylenediaminetetraacetic acid (K_3_EDTA) tubes.

## Methods

This was an international, blinded, prospective cohort study enrolling patients presenting to two adult large EDs at the Pitié-Salpêtrière APHP-Sorbonne Université hospital in Paris (LPS), France, and the Hospital Universitari Germans Trias i Pujol in Badalona, Spain (UHG). The study assessed MDW’s ability to detect the development of sepsis in consecutive adult patients (18–89 years) presenting to the ED and who had a CBC-DIFF ordered. The study was registered in ClinicalTrials.gov (NCT03588325) and approved by the ethical committees of the respective participating countries.

### Patients

**Fig. 1:**
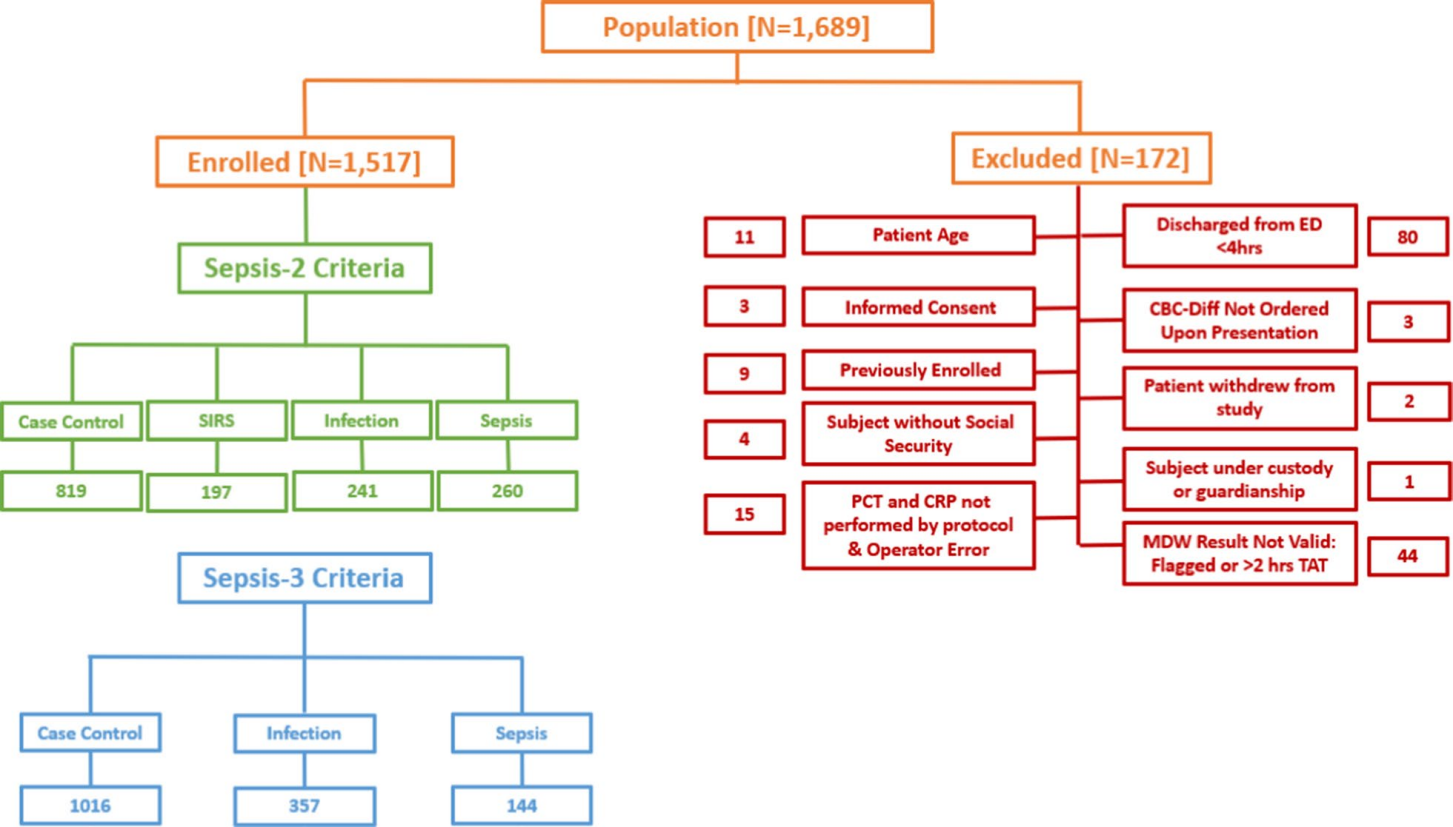
Flow diagram describing patient screening and enrollment. MDW, monocyte distribution width; SIRS, systemic inflammatory response syndrome; CBC-DIFF, complete blood count with differential; ED, Emergency Department; PCT, procalcitonin; CRP, C-reactive protein; TAT, Turnaround time

The study prospectively enrolled consenting adults (over 18-year-old) presenting to the ED, whose initial evaluation included a CBC-DIFF. Exclusion criterion is as shown in Fig. 1. Emergency physicians were free to order necessary tests according to the standard of care. Treating physicians were blinded to MDW, PCT and CRP values obtained by protocol, but received PCT and/or CRP results they ordered as current practice.

Both PCT and CRP are used routinely at LPS, and CRP is used routinely at UHG.

### Blood sampling

Briefly, patients who were identified to have a CBC-DIFF ordered by the physician were asked to participate. After informed consent, baseline blood draws were performed. An additional K_3_EDTA tube was drawn together with a sample for PCT and CRP measurement as well as routine blood tests at the discretion of the treating physician. Samples for MDW measurement were analyzed on a UniCel DxH 900 analyzer (Beckman Coulter, Inc., Brea, CA) with version 1.0.0.329 software within two hours of collection. This instrument measures specific cell volume variables and the distribution of cell volumes within a group of white blood cells (WBC). Quality control was performed daily with COULTER 6C Plus Cell Control to monitor the DxH 900 system performance. COULTER LATRON CP-X Control was used as part of the daily quality control procedure to monitor volume, conductivity and light scatter measurements. PCT and CRP concentrations were measured on Cobas analyzer (Roche Diagnostics, Meylan, France) at LPS, while UHG utilized Liaison XL (DiaSorin, Saluggia, Italy) and AU5800 (Beckman Coulter, Inc, Brea, CA, USA) analyzers for PCT and CRP measurements, respectively.

### Clinical data, follow-up, blinding and adjudication

Clinical data at presentation, including past medical history, assessment of vital signs, symptoms, SIRS criteria [12], qSOFA [13] and SOFA scores [14], microbiological testing and treatments were recorded on an electronic case report form and patients followed up for at least 12 h. The clinical research team and physician adjudicators were blinded to MDW results at the time of clinical data entry and during assignment of the patients to a clinical category. Adjudicators were also blinded to the results of PCT and CRP if not ordered by the treating ED physician.

Study subjects were classified by at least two independent physician adjudicators at each site. Discordances were arbitrated by a third independent physician. Adjudicators categorized subjects based on the “Sepsis-2” consensus criteria [15], such as non-SIRS or case controls (zero or one SIRS criterion and no infection), SIRS (≥ 2 SIRS criteria and no infection), infection (suspected or confirmed infection with 0—1 SIRS criteria), sepsis (infection plus ≥ 2 SIRS) (including sepsis [no organ failures], severe sepsis [sepsis with one or more organ failures] and septic shock [sepsis with refractory hypotension]). Adjudicated categories per Sepsis-3 criteria [16] included controls, infection and sepsis (based on SOFA score criteria). In order to characterize sepsis as being present upon ED admission, sepsis criteria had to be fulfilled within 12 h of the initial CBC-DIFF in patients with suspected infection (as reflected by initiation of diagnostic infection workup) and adjudication was based on the retrospective chart review of tests ordered and clinical data available within the first 12 h of ED presentation. If no infection work-up was performed within 12 h, or if the adjudicator believed that the infection work-up showed no evidence of infection, the patient was categorized as “not infected” or SIRS by the adjudicator. The test results were extracted from the medical records 7–10 days later, including cultures, molecular tests (e.g., polymerase chain reaction), antigens tests (immunoassay) and relevant imaging. A subgroup, consisting of subjects for whom no CRP or PCT was ordered by the emergency physician, was identified (post study) and defined as having low sepsis pretest probability.

### Statistical analysis

General descriptive statistics and box plots were calculated for cell population variables. The sample size calculations were based on 95% two-sided confidence interval and 80% power. Proc power of SAS 9.3 was used for calculating sample size based on the approach presented in Johnson et al. [17] A target sensitivity of 75% with the lower limit of the 95% two-sided confidence interval of 65% and a target specificity of 70% with the lower limit of the 95% two-sided confidence interval of 65% were assumed. A minimum of 189 septic subjects and a minimum of 817 non-septic subjects were needed from both sites combined.

Diagnostic ability was evaluated in terms of the area under the ROC curve (AUC), sensitivity, specificity, positive predictive value (PPV), negative predictive value (NPV), positive and negative likelihood ratios, along with their 95% confidence intervals (CIs). The score approach was used to calculate CI for sensitivity, specificity, PPV and NPV. Differences in AUC were used to demonstrate the added value of MDW in comparison with WBC alone, calculated using a one-predictor variable logistic model with WBC and a two-predictor variables logistic model with both WBC and MDW, as the predictor, and using sepsis status as the response. A similar approach was applied to analyze the diagnostic ability of PCT and CRP, and the combination with WBC and MDW for sepsis detection. AUC comparisons along with their CI were calculated as described by DeLong et al. [18] SAS 9.4 (SAS Institute, Cary, NC) statistical program was used for data analyses.

### Cutoff determination

PCT cutoff of > 0.25 µg/mL was based on the literature. The determination of the cutoff for CRP (> 22 mg/L) was based on the Youden index using the data from this clinical study. The proposed MDW cutoff of 21.5 units for K_3_EDTA was based on previously established MDW cutoff (in K_2_EDTA) for sepsis identification in the ED and internal testing demonstrating a shift of 1.5 units for blood specimens collected in K_3_EDTA versus K_2_EDTA (data not shown). This study validates the new MDW cutoff of 21.5 for K_3_EDTA.

### Probabilities and odds ratios

Identification of sepsis in the ED relies on the physician’s ability to assess probability of disease based on presenting symptoms. To reflect this approach, we analyzed the probability and likelihood ratios for sepsis based upon the values of MDW in combination with SIRS or qSOFA parameters determined during the initial patient encounter in the ED (typically within the first 2 h of ED admission). Predicted probability of a positive sepsis diagnosis was calculated from the positive likelihood ratios (LRs +) as previously described [19, 20]. In this approach, predicted sepsis probability after receiving test results, or posttest probability *P*_1_, is based on an estimated pretest probability *P*_0_ and LR + and is calculated as:

$$P_{1} = \frac{{P_{1} \times {\text{LR}} + }}{{(1 - P_{0} + P_{0} \times {\text{LR }} +)}}$$

where *P*_0_ is the sepsis prevalence of the study cohort. The odds ratios (ratios of posttest probabilities) for sepsis diagnosis between parameter combinations with abnormal and normal MDW values were calculated based on prevalence of sepsis-2 or sepsis-3 for the study population.

## Results

As shown in the flow diagram of the study (Fig. 1), from August 2, 2018, through June 27, 2019, 1,689 patients were screened and enrolled, and 172 were excluded due to patient not meeting inclusion criteria, inadequate sample collection or screening errors. The final analysis included 1,517 patients (LPS 837, UHG 680). Demographic features are shown in Table 1. There were 837 men and 680 women, median age 64 (IQ 47–76 years). Using Sepsis-2 criteria, 819 patients (54%) were categorized as non-SIRS/non-sepsis (case controls) while 260 (17.1%) had sepsis, including 38 with severe sepsis. According to Sepsis-3 criteria, 1,016 patients (67%) were adjudicated as case controls and 144 patients (9.5%) had sepsis. Eighty-five percent of Sepsis-2 patients had at least one microbiological test performed, of which 58% were positive. Bacterial cultures grew with Gram-positive species in 16 patients, Gram-negative in 62 patients and mixed gram stain in 10. These cultures were 41 from urine, 24 from blood, 14 from sputum and 9 from other sources (Additional file 2: Sup. Table 5). Five hundred and fifty-three patients (36.5%) fell in the subgroup of the low pretest probability of sepsis (no CRP or PCT ordered by the emergency physicians).

**Table 1:** Demographic data. Data were available for all the patients, unless indicated by N. Quantitative data are expressed as Medians (interquartile range)

	LPS	UHG	Sites combined	Sepsis-2 N = 260	Sepsis-3 N = 144
Clinical characteristics					
Female (No. %)	404 (48.3%)	276 (40.6%)	680 (44.8%)	101 (38.9%)	46 (31.9%)
Male (No. %)	433 (51.7%)	404 (59.4%)	837 (55.2%)	159 (61.2%)	98 (68.1%)
Age, years	57 (39–71)	72 (60–80)	64 (47–76)	66 (51–77)	72 (61–80)
Temperature (°C) N = 1506	36.6 (36.2–37.0)	36.2 (36.0–36.7)	36.5 (36.0–36.9)	37.1 (36.5–38.2)	36.8 (36.2–38.0)
Heart rate (beats/min)	85 (73–100)	82 (70–98)	84 (71–99)	100 (92–110)	93 (75–106)
Respiratory (breaths/min) N = 865	20 (16–22)	18 (16–24)	19.0 (16–24)	22 (18–26)	23 (18–26)
Immunosuppression	38 (4.5%)	101 (14.9%)	139 (9.2%)	38 (14.6%)	22 (15.3%)
Laboratory parameters					
WBC (1 × 10^3^/μL)	8.33 (6.24–10.94)	9.17 (6.86–11.89)	8.71 (6.50–11.34)	13.02 (9.02–16.28)	10.82 (8.27–15.03)
PMN (1 × 10^3^/μL)	5.80 (3.88–8.44)	6.74 (4.73–9.42)	6.27 (4.23–8.90)	10.65 (7.09–14.04)	8.99 (6.15–13.18)
EO (1 × 10^3^/μL)	0.07 (0.02–0.15)	0.07(0.02–0.14)	0.07 (0.02–0.14)	0.02 (0.01–0.06)	0.02 (0.0–0.06)
MDW (U)	19.28 (17.85–21.68)	21.09 (19.19–24.03)	20.15 (18.22–22.79)	23.92 (21.54–26.94)	24.61 (22.33–28.47)
PCT (ng/mL)	0.04 (0.02–0.10)	0.06 (0.02–0.24)	0.05 (0.02–0.15)	0.21 (0.07–0.79)	0.34 (0.13–1.54)
CRP (mg/L)	5.22 (0.85–31.76)	18.30 (4.75–82.50)	10.10 (1.91–52.89)	82.50 (36.90–185.50)	95.35 (44.13–197.98)
CREAT (μmol/L) N = 1489	76.00 (64.00–95.00)	80.40 (62.80–113.20)	78.00 (63.60–102.00)	84.00 (65.40–123.00)	115.35 (74.30–186.50)

LPS, Pitié-Salpêtrière APHP-Sorbonne Université Hospital; UHG, Hospital Universitari Germans Trias i Pujol; WBC, white blood count; PMN, polymorphonuclear; EO, eosinophils; MDW, monocyte distribution width; PCT, procalcitonin; CRP, C-reactive protein; CREAT, creatinine

### MDW performance for sepsis detection

**Fig. 2:**
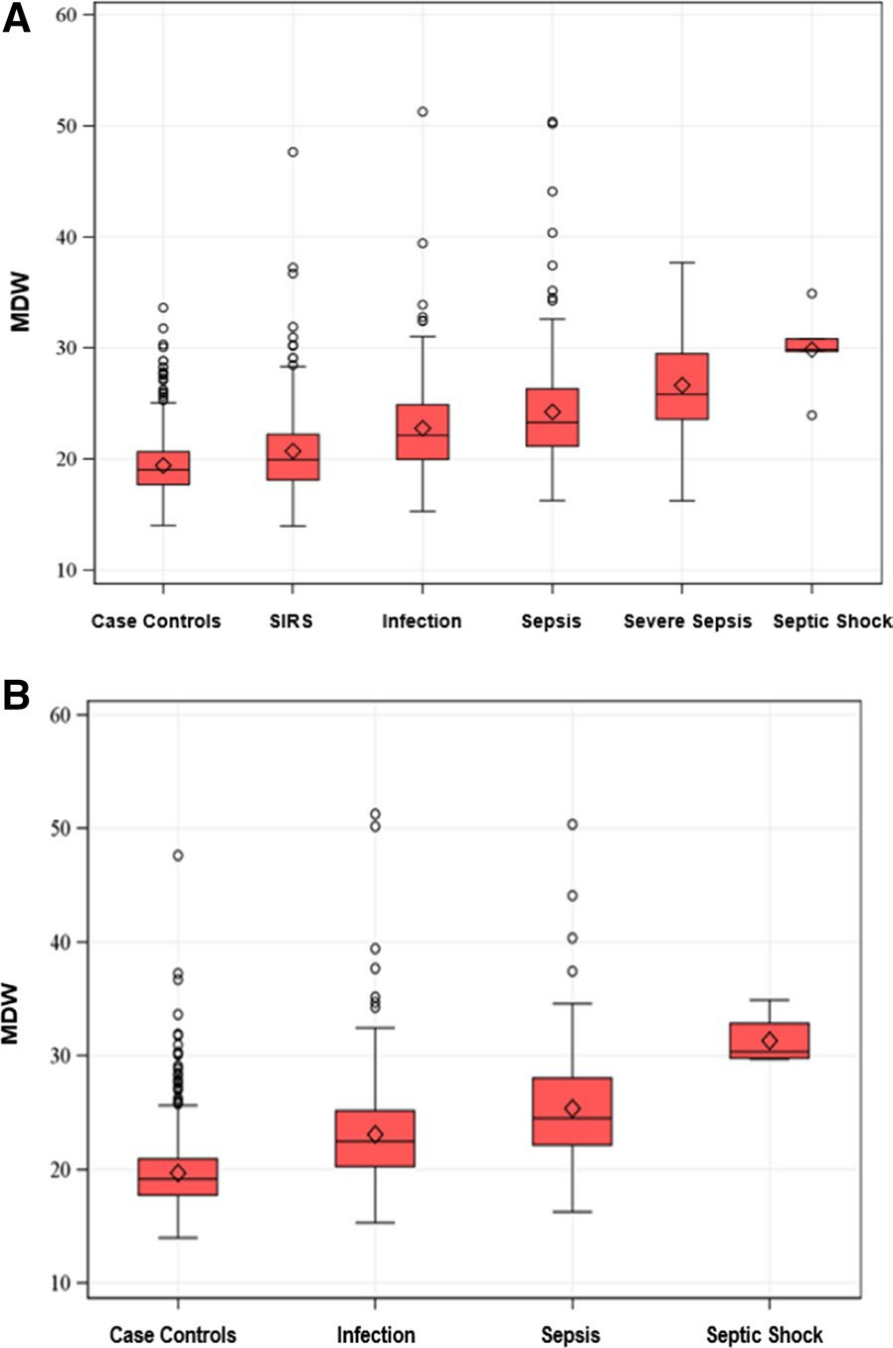
Box plots of MDW baseline values conforming to sepsis classification by Sepsis-2 criteria (**A**) and Sepsis-3 criteria (**B**). MDW, monocyte distribution width; SIRS, systemic inflammatory response syndrome

**Fig. 3:**
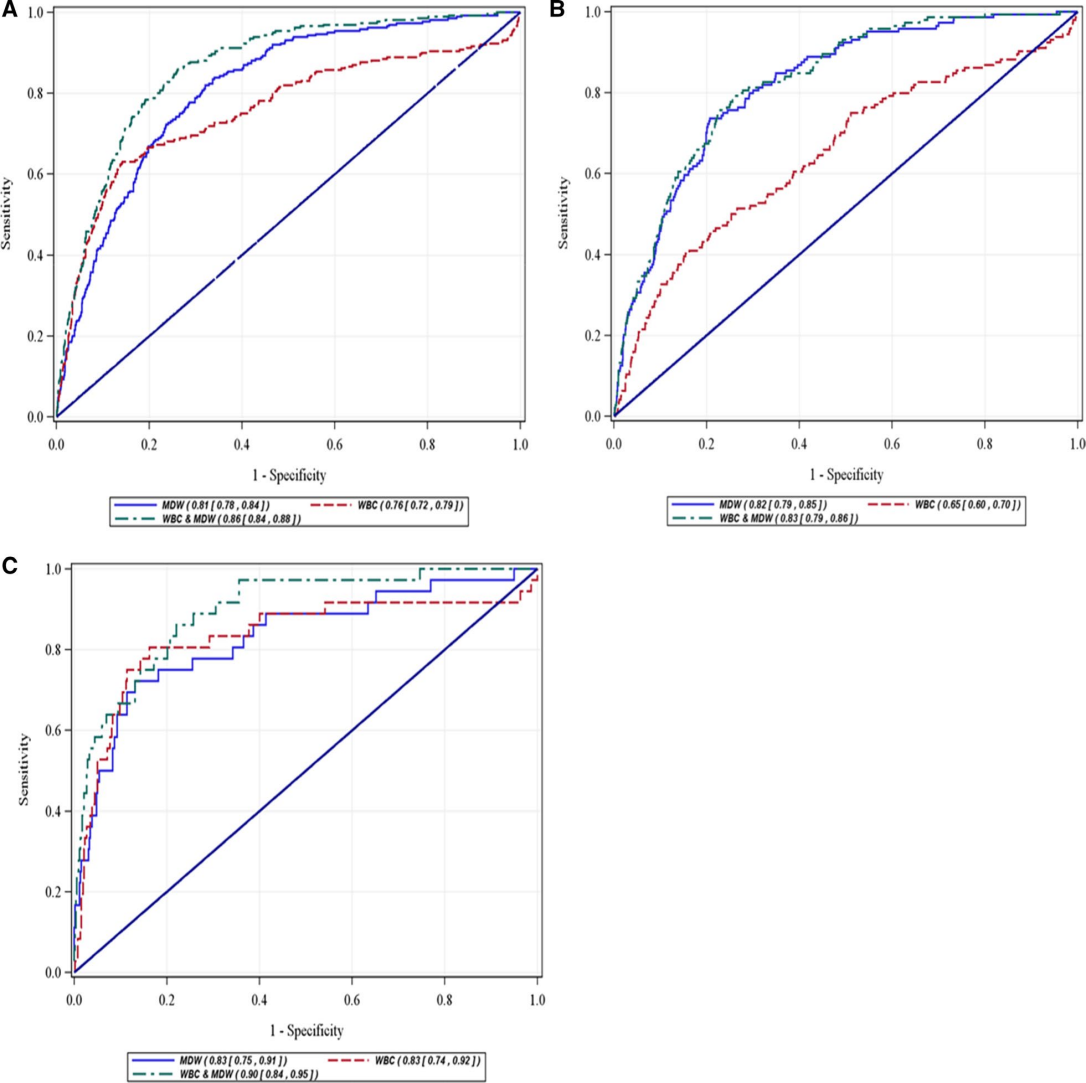
MDW, WBC and MDW + WBC performance for sepsis diagnosis. **A** Sepsis-2. **B** Sepsis-3. **C** Sepsis-2, low pretest probability population (CRP or PCT not ordered by emergency physician). MDW, monocyte distribution width; WBC, white blood count

Figure 2A, B illustrates box whisker plots for a single baseline MDW measurement according to Sepsis-2 (A) or Sepsis-3 (B) criteria showing increases in MDW values with infection and sepsis severity, regardless of sepsis criteria used. In K_3_EDTA tubes, the MDW cutoff of 21.5 provides an optimum diagnostic power by balancing the ability to detect positive sepsis patients (sensitivity) and case controls (specificity). The area under the receiver operating characteristic (AUROC) curves for Sepsis-2 was 0.81 [95% confidence interval 0.78–0.84] for MDW vs 0.76 [0.72–0.79] for WBC and 0.86 [0.84–0.88] when combined with WBC (Fig. 3a). For Sepsis-3, MDW AUROC was 0.82 [0.79–0.85] compared to 0.65 [0.60–0.70] for WBC (Fig. 3b). Adding WBC to MDW assessment added no value for Sepsis-3 diagnosis. For the subgroup of patients with low sepsis pretest probability, AUROC for Sepsis-2 was 0.83 [0.75–0.91] for MDW alone and 0.90 [0.84–0.95] when combined with WBC (Fig. 3c).

### MDW added value for sepsis detection

**Fig. 4:**
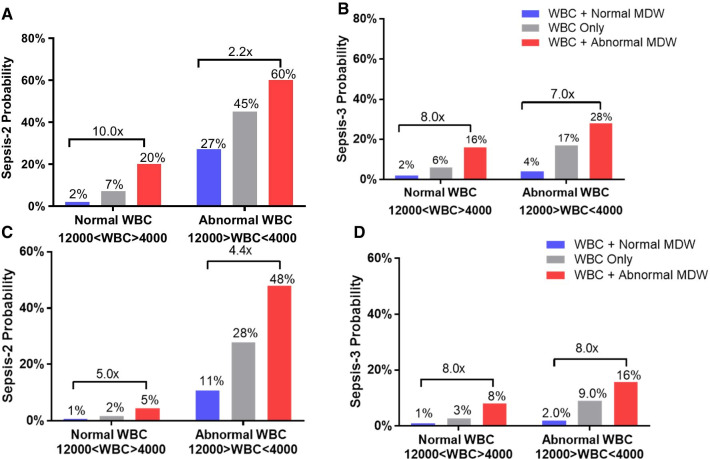
Added value of MDW on sepsis posttest probabilities according to WBC range at presentation. **A** Sepsis-2 (sepsis pretest probability = 0.17). **B** Sepsis-3 (pretest 0.09). **C** Sepsis-2, low pretest probability population (CRP or PCT not ordered by emergency physician. Sepsis pretest probability = 0.065). Cutoff: WBC < 4,000/mm^3^ or WBC > 12,000, MDW > 21.5, and PCT > 0.25 µg/L. **D** low pretest probability population per Sepsis-3 (pretest = 0.043). MDW, monocyte distribution width; WBC, white blood count; PCT, procalcitonin; CRP, C-reactive protein

The value of MDW significantly modified the observed posttest probability of sepsis vs. the baseline probability regardless of the sepsis definitions, specific clinical risk scores (SIRS and qSOFA) and values of WBC, with abnormal (> 21.5) MDW increasing and normal MDW decreasing the posttest probability. Compared to normal MDW, abnormal MDW increased the odds of Sepsis-2 by a factor of 5.5 [4.2–7.1, 95% CI] and the odds of Sepsis-3 by 7.6 [5.1–11.3, 95% CI] (Table 2), with highest increases occurring in patients with low SIRS and qSOFA scores (Table 2 and Additional file 3: Sup. Figure 6). Abnormal MDW increased the probability of Sepsis-2 for all ranges of WBC including in patients with low pretest probability with similar effect observed using Sepsis-3 adjudication (Fig. 4a–d and Additional file 4: Sup. Figure 7).

**Table 2:** Added value of MDW to SIRS criteria for Sepsis-2 diagnosis (pretest = 0.17), and to qSOFA for Sepsis-3 diagnosis (pretest = 0.9)

Sepsis 2 & SIRS	No SIRS	1 SIRS	2 SIRS	3 SIRS	4 SIRS	Total
Total patients	515	594	271	117	20	1517
Sepsis patients (#)	4	21	141	81	13	260
Sepsis probability, MDW unknown	0.8%	3.5%	51.8%	69.0%	64.8%	17.0%
Sepsis probability, MDW normal	0.3%	1.7%	31.1%	37.6%	28.4%	6.6%
Sepsis probability, MDW abnormal	2.4%	7.8%	71.2%	83.6%	84.5%	36.1%
Sepsis odds (MDW abnormal/normal) [95% CI]	9.3	4.7	2.3	2.2	3.0	5.5[4.2–7.1]

Sepsis 3 & qSOFA	No qSOFA	1 qSOFA	2 qSOFA	3 qSOFA		Total
Total patients	1136	347	33	1		1517
Sepsis patients (#)	53	72	18	1		144
Sepsis probability, MDW unknown	4.4%	19.8%	53.1%			9.0%
Sepsis probability, MDW normal	1.2%	8.1%	28.8%			2.7%
Sepsis probability, MDW abnormal	12.0%	31.9%	63.9%			20.6%
Sepsis odds (MDW abnormal/normal) [95% CI]	10.1	3.9	2.2			7.6 [5.1–11.3]

MDW, monocyte distribution width; SIRS, systemic inflammatory response syndrome; qSOFA, quick Sequential Organ Failure Assessment; CI, Confidence Interval

### Comparison of MDW with PCT and CRP performance for sepsis detection

The respective performance of PCT and CRP and combined with WBC and MDW is represented in Table 3. Overall, the diagnostic ability of MDW combined with WBC was similar to CRP alone (AUROC: 0.86 [0.84–0.88] *vs.* 0.85 [0.83–0.87], respectively) and performed better than PCT alone (AUROC: 0.78 [0.75–0.81]). Adding either CRP or PCT to MDW/WBC analysis did not improve the AUC. The sensitivity and specificity of using MDW and/or WBC in combination, based on their individual cutoffs, is shown in Additional file 1: Sup. Table 4. If either parameter was abnormal (“or”) the sensitivity was > 90%, if both parameters were abnormal (“and”) then as expected the specificity of the combination of MDW + WBC was high (> 90%).

**Table 3:** Comparison of MDW with WBC, PCT and CRP performance for sepsis detection Cutoff: WBC < 4,000/mm^3^ or WBC > 12,000/mm^3^, MDW > 21.5, PCT > 0.25 µg/L, CRP > 22 mg/L)

Sepsis-2							
Parameter	Sensitivity [95%CI]	Specificity [95%CI]	PPV [95%CI]	NPV [95%CI]	LR + [95%CI]	LR − [95%CI]	AUC [95%CI]
MDW	0.75 [0.69–0.80]	0.73 [0.70–0.75]	0.36 [0.32–0.40]	0.93 [0.92–0.95]	2.76 [2.46–3.09]	0.34 [0.28–0.43]	0.81 [0.78–0.84]
WBC	0.69 [0.63–0.74]	0.83 [0.81–0.85]	0.45 [0.40–0.50]	0.93 [0.91–0.94]	4.00 [3.50–4.60]	0.40 [0.30–0.50]	0.76 [0.72–0.79]
PCT	0.45 [0.39–0.51]	0.88 [0.86–0.90]	0.44 [0.38–0.50]	0.89 [0.87–0.90]	3.80 [3.10–4.65-]	0.62 [0.56–0.70]	0.78 [0.75–0.81]
CRP	0.85 [0.80–0.88]	0.72 [0.70–0.75]	0.39 [0.35–0.43]	0.96 [0.94–0.97]	3.06 [2.76–3.39]	0.21 [0.16–0.28]	0.85 [0.83–0.87]
MDW + WBC							0.86 [0.84–0.88]
MDW + PCT							0.81 [0.78–0.84]
MDW + CRP							0.85 [0.82–0.87]
MDW + WBC + PCT							0.86 [0.84–0.89]
MDW + WBC + CRP							0.87 [0.85–0.89]
**Sepsis-3**							
MDW	0.81 [0.73–0.86]	0.69 [0.67–0.72]	0.22 [0.18–0.25]	0.97 [0.96–0.98]	2.63 [2.35–2.94]	0.28 [0.20–0.39]	0.82 [0.79–0.85]
WBC	0.49 [0.41–0.57]	0.77 [0.74–0.79]	0.18 [0.15–0.22]	0.94 [0.92–0.95]	2.10 [1.70–2.50]	0.70 [0.60–0.80]	0.65 [0.60–0.70]
PCT	0.60 [0.52–0.68]	0.87 [0.85–0.89]	0.33 [0.27–0.39]	0.95 [0.94–0.96]	4.63 [3.83–5.60]	0.46 [0.37–0.56]	0.84 [0.80–0.87]
CRP	0.89 [0.83–0.93]	0.68 [0.66–0.71]	0.23 [0.19–0.26]	0.98 [0.97–0.99]	2.79 [2.53–3.07]	0.16 [0.10–0.26]	0.85 [0.82–0.87]
MDW + WBC							0.83 [0.79–0.86]
MDW + PCT							0.82 [0.79–0.86]
MDW + CRP							0.85 [0.82–0.88]
MDW + WBC + PCT							0.83 [0.80–0.86]
MDW + WBC + CRP							0.85 [0.82–0.87]

MDW, monocyte distribution width; WBC, white blood count; PCT, procalcitonin; CRP, C-reactive protein; PPV, positive predictive value; NPV, negative predictive value; LR + : positive likelihood ratio. LR-, negative likelihood ratio; AUC: area under the ROC curve; CI confidence interval

**Fig. 5:**
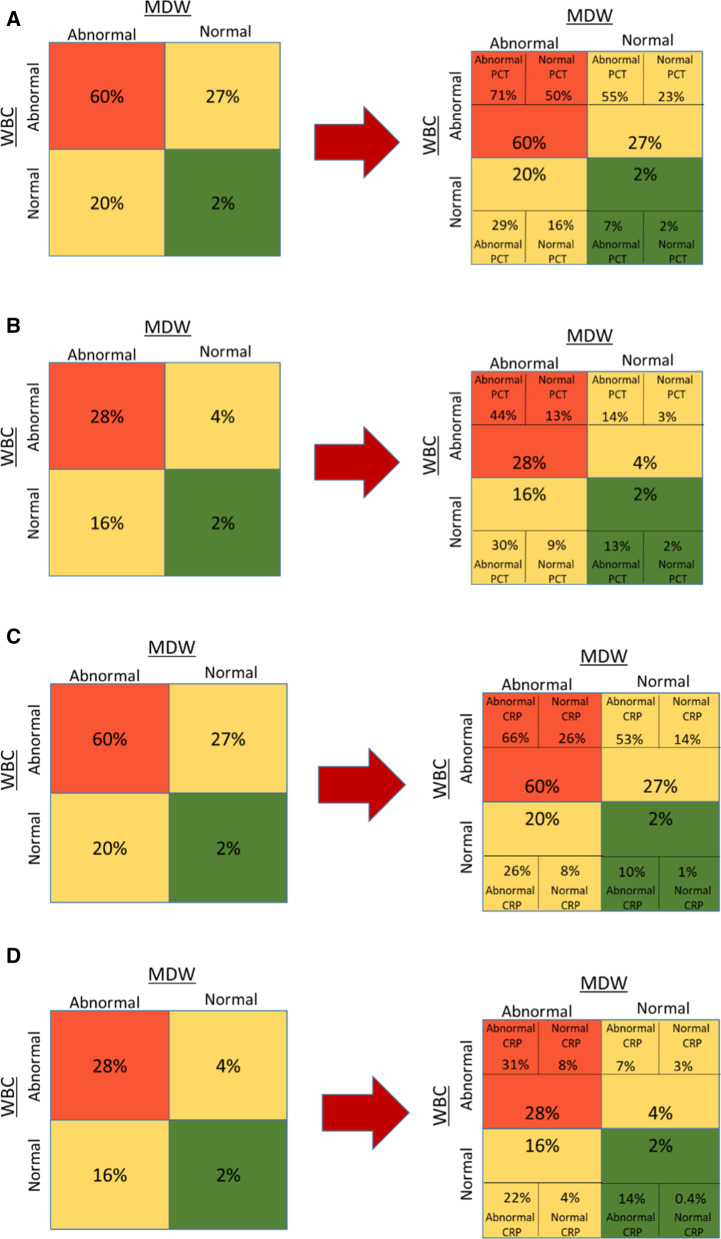
Sequential assessment of sepsis probabilities according to WBC, MDW and PCT results. Cutoff: WBC < 4,000/mm^3^ or WBC > 12,000/mm^3^, MDW > 21.5, PCT > 0.25 µg/L, CRP > 22 mg/L). Pretest probabilities were 0.17 for sepsis-2 and 0.09 for sepsis-3. **A** PCT by Sepsis-2. **B** PCT by Sepsis-3. **C** CRP by Sepsis-2. **D** CRP by Sepsis-3. MDW, monocyte distribution width; WBC, white blood count; PCT, procalcitonin; CRP, C-reactive protein

Figure 5a–d represents the posttest sepsis probabilities of the sequential results of baseline WBC, MDW and PCT or CRP tests at their specified cutoffs. When WBC is normal (in the 4,000–12,000/mm^3^ range), an MDW value > 21.5 is associated with a tenfold increase of Sepsis-2 probability (compared to MDW normal), and an eightfold increase of Sepsis-3. Similarly, when WBC is abnormal (< 4,000 or > 12,000/mm^3^) and MDW > 21.5, the probability of Sepsis-2 is twice the one when MDW is normal, and sevenfold for Sepsis-3.

When WBC is normal but MDW is abnormal (> 21.5), addition of PCT increases probability of sepsis to the same level as when both (WBC, MDW)parameters were abnormal, threefold above the pretest probability (see Fig. 5b, PCT > 0.25 and MDW > 21.5 and WBC normal sepsis probability is 30%). A similar pattern was observed with CRP. Similarly, when WBC is abnormal but MDW is normal, addition of PCT > 0.25 µg/L increases the Sepsis-2 probability approximately threefold comparable to when both WBC and MDW are both abnormal.

## Discussion

In this multicenter clinical trial conducted in two large European EDs, we confirmed the performance of MDW as an early sepsis biomarker with an AUC of 0.81 [0.78–0.84] and 0.86 [0.84–0.88] when combined with WBC for Sepsis-2 criteria. These results are in accordance with those of two recent North American studies which reported very similar performance of MDW (AUC 0.79 [0.73–0.84] and 0.79 [0.76–0.82], respectively) [8, 9] which argues for the robustness and reproducibility of this new biomarker in different populations. At an optimal cutoff of 21.5 in K_3_EDTA tubes, a sensitivity of 75% [69–80, 95% CI] and specificity of 73% [70–75, 95% CI] were observed which again agrees well with previous studies in North America and demonstrates that the anticoagulant does not affect the diagnostic accuracy of the biomarker. Moreover, we reported that MDW combined with WBC was particularly useful to detect sepsis in a low pretest probability subgroup of patients (AUC: 0.90 [0.84–0.95]), that is patients for whom no sepsis biomarker was ordered by the emergency physicians upon presentation. MDW may be displayed routinely as part of CBC with differential for most of the patients who have blood drawn in the ED and thus provides an added value to the early clinical evaluation. Indeed, early sepsis detection is warranted to initiate specific goal-directed therapy bundles [5]; hence, the systematic SIRS criteria and qSOFA score calculation are highly recommended. However, both SIRS and qSOFA lack sensitivity and specificity and may falsely lead to over or under suspicion of sepsis [4, 6, 21, 22]. In the present study, we confirmed that MDW provided an added diagnostic power to SIRS and qSOFA variables, with an overall odd of 5.5 and 7.6 for Sepsis-2 and Sepsis-3, respectively, when MDW > 21.5 (Table 2 and Fig. 5). As recently published by Crouser, et al., because MDW value can be displayed with CBC results early in the ED’s course of the patient, it may be considered as a fifth SIRS or fourth qSOFA criteria [10].

MDW combined with WBC had a similar AUC compared to CRP (0.85 [0.82–0.87]) and performed better than PCT (0.78 [0.75–0.81]) (Table 3). To our knowledge, this was the first report of the comparison between MDW, PCT and CRP in the emergency department, in contrast to the study by Polilli, et al., who reported nearly overlapping AUC for MDW and PCT (0.87 *vs. 0.88*, respectively) in a cohort of hospitalized patients for suspected infection or sepsis [11]. The performances of PCT and CRP we observed were much higher than what had been reported to date in the literature, with AUCs ranging from 0.64 to 0.79 and 0.57 to 0.79, respectively [23–27]. The first explanation of the discrepancy is that our main inclusion criterion (ED patients for whom initial evaluation included a CBC-DIFF) resulted in a less selected patient population than the criteria of previous studies (patients with a suspicion of infection or sepsis). The sepsis prevalence observed in current study (Table 1) was higher than the mean prevalence in ED population [28], but much lower than what is reported in more selected populations [23, 24]. For example, Lungström, et al., reported a Sepsis-2 prevalence of 42% in a cohort of 1,572 EDs patients with a suspicion of infection, along with PCT and CRP AUCs of O.64 [0.61–0.67] and 0.57 [0.54–0.60], respectively, whereas Uusitalo-Seppälä, et al., reported a Sepsis-2 prevalence of 57% and AUCs of 0.77 [0.71–0.84] and 0.60 [0.51–0.69], respectively, for severe sepsis detection [23, 24]. The second hypothesis was that although the adjudicators were blinded to PCT and CRP results performed by protocol, they had access to PCT and CRP values obtained as standard of care, which may have contributed to an overestimation of the performance of these two sepsis biomarkers in our study.

On first analysis, our data did not support the addition of PCT and CRP testing to WBC plus MDW in the ED. However, as illustrated in Fig. 5, a sequential approach with WBC plus MDW as a systematic screening test, followed by PCT or CRP measurement in cases of discordant results (WBC abnormal–MDW normal, or WBC normal–MDW abnormal) may represent a pragmatic perspective.

We acknowledge several limitations of the study. First, as there is no gold standard for the diagnosis of sepsis, we cannot exclude the possibility of some misclassification of patients that limits or biases the accuracy of the biomarkers. However, we tried to limit this risk by adjudicating all cases using two independent adjudicators and arbitration by a third physician in case of disagreement. Second, as discussed above, the adjudicators were not blinded to PCT and CRP values ordered as standard of care, which may have contributed to an overestimation of both the prevalence of sepsis and the performance of these biomarkers. Similarly, sepsis biomarker ordering as a standard of care was different by sites (PCT and CRP in LPS, CRP only in UHG). We think that this current practice discrepancy rather enhances the validity of the combined sites results than biases it. Finally, the overall sepsis prevalence we reported was higher than the usual prevalence in EDs, arguing for a part of screening selection bias toward sepsis patients at inclusion.

## Conclusion

MDW in combination with WBC has the diagnostic accuracy to detect sepsis, particularly when assessed in patients with lower pretest sepsis probability. We suggest the use of MDW as a systematic screening test, used together with SIRS criteria and qSOFA score to improve the accuracy of sepsis diagnosis [29, 30] in the emergency department. The place of CRP or PCT thereafter remains to be determined.

## Supplementary Information

**Additional file 1**. Combining MDW and WBC to improve sepsis detection per Sepsis-2 and Sepsis-3 criteria. Abbreviations: MDW, monocyte distribution width; WBC, white blood count.

**Additional file 2**. Microbiological Tests per Sepsis-2 and Sepsis-3 Criteria.

**Additional file 3**. Added value of MDW to SIRS criteria for Sepsis2 diagnosis (pre-test = 0.17) and to qSOFA for Sepsis-3 diagnosis (pre-test = 0.09). Abbreviations: MDW, monocyte distribution width; SIRS, systemic inflammatory response syndrome; qSOFA, quick Sequential Organ Failure Assessment.

**Additional file 4**. Added value of MDW on sepsis post-test probabilities according to WBC range at presentation. A: Sepsis-2 (sepsis pre-test probability = 0.17). B: Sepsis-3 (pre-test 0.09). C: Sepsis-2, low pre-test probability population (pre-test = 0.065) D: low pre-test probability population per Sepsis-3 (pre-test = 0.043).

## Data Availability

On request at Beckman Coulter, Inc.
