# Supplementary material for: Monocyte distribution width (MDW) performance as an early sepsis indicator in the emergency department: comparison with CRP and procalcitonin in a multicenter international European prospective study
Source: Crit Care. 2021 Jun 30;25:227. doi: 10.1186/s13054-021-03622-5 (PMC8247285; doi:10.1186/s13054-021-03622-5)
Supplement: Supplementary file 1 — Additional file 1. Combining MDW and WBC to improve sepsis detection per Sepsis-2 and Sepsis-3 criteria. Abbreviations: MDW, monocyte distribution width; WBC, white blood count. [file 13054_2021_3622_MOESM1_ESM.docx]

**Additional file 1:** Combining MDW and WBC to improve sepsis detection per Sepsis-2 and Sepsis-3 criteria.

Abbreviations: MDW, monocyte distribution width; WBC, white blood count; PCT, procalcitonin; CRP, C-reactive Protein; PPV, Positive predictive value; NPV, negative predictive value; LR+: Positive likelihood ratio. LR-, Negative likelihood ratio; AUC: area under the ROC curve; CI Confidence Interval
