# Supplementary material for: Monocyte distribution width (MDW) performance as an early sepsis indicator in the emergency department: comparison with CRP and procalcitonin in a multicenter international European prospective study
Source: Crit Care. 2021 Jun 30;25:227. doi: 10.1186/s13054-021-03622-5 (PMC8247285; doi:10.1186/s13054-021-03622-5)
Supplement: Supplementary file 2 — Additional file 2. Microbiological Tests per Sepsis-2 and Sepsis-3 Criteria. [file 13054_2021_3622_MOESM2_ESM.docx]

**Additional file 2:** Microbiological Tests per Sepsis-2 and Sepsis-3 Criteria

1. All Microbial Tests per Sepsis-2 Criteria

1. Specimen Source for Bacterial Cultures per Sepsis-2 Criteria

1. Microbial Tests per Sepsis-3 Criteria All
