# Supplementary material for: Monocyte distribution width (MDW) performance as an early sepsis indicator in the emergency department: comparison with CRP and procalcitonin in a multicenter international European prospective study
Source: Crit Care. 2021 Jun 30;25:227. doi: 10.1186/s13054-021-03622-5 (PMC8247285; doi:10.1186/s13054-021-03622-5)
Supplement: Supplementary file 3 — Additional file 3. Added value of MDW to SIRS criteria for Sepsis2 diagnosis (pre-test = 0.17) and to qSOFA for Sepsis-3 diagnosis (pre-test = 0.09). Abbreviations: MDW, monocyte distribution width; SIRS, systemic inflammatory response syndrome; qSOFA, quick Sequential Organ Failure Assessment. [file 13054_2021_3622_MOESM3_ESM.docx]

**Additional file 3:** Added value of MDW to SIRS criteria for Sepsis2 diagnosis (pre-test =0.17) and to qSOFA for Sepsis-3 diagnosis (pre-test =0.09).

Abbreviations: MDW, monocyte distribution width; SIRS, systemic inflammatory response syndrome; qSOFA, quick Sequential Organ Failure Assessment

**A. SIRS criteria and Sepsis-2**

**
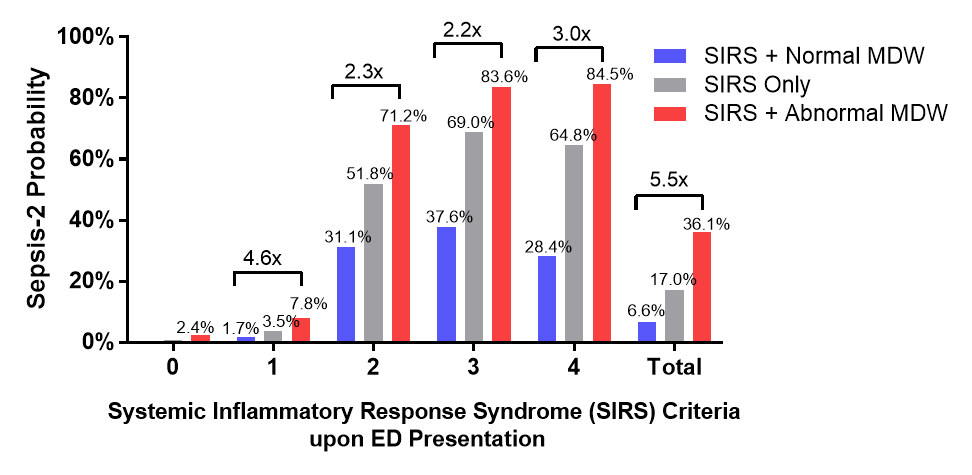
**

**B. qSOFA score and Sepsis-3**

**
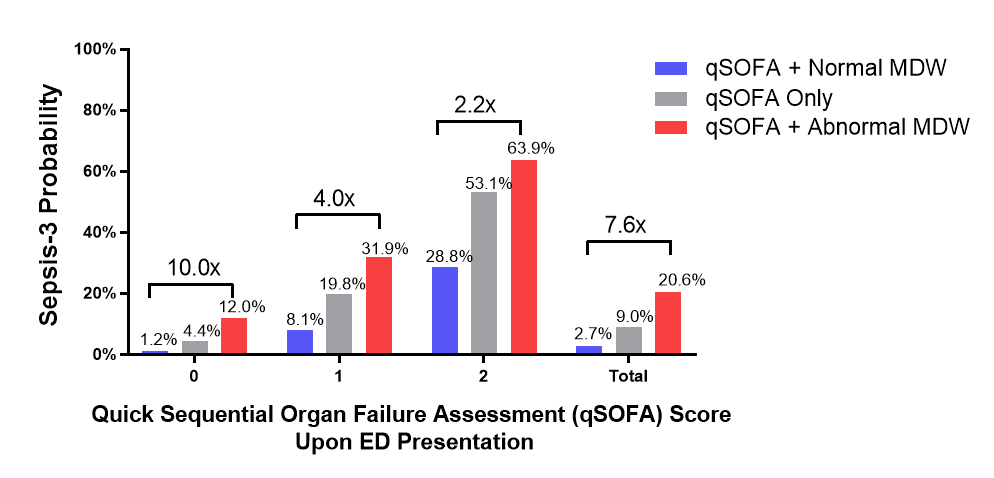
**
