# Supplementary material for: Monocyte distribution width (MDW) performance as an early sepsis indicator in the emergency department: comparison with CRP and procalcitonin in a multicenter international European prospective study
Source: Crit Care. 2021 Jun 30;25:227. doi: 10.1186/s13054-021-03622-5 (PMC8247285; doi:10.1186/s13054-021-03622-5)
Supplement: Supplementary file 4 — Additional file 4. Added value of MDW on sepsis post-test probabilities according to WBC range at presentation. A: Sepsis-2 (sepsis pre-test probability = 0.17). B: Sepsis-3 (pre-test 0.09). C: Sepsis-2, low pre-test probability population (pre-test = 0.065) D: low pre-test probability population per Sepsis-3 (pre-test = 0.043). [file 13054_2021_3622_MOESM4_ESM.docx]

**Additional file 4:** Added value of MDW on sepsis post-test probabilities according to WBC range at presentation. A: Sepsis-2 (sepsis pre-test probability= 0.17). B: Sepsis-3 (pre-test 0.09). C: Sepsis-2, low pre-test probability population (pre-test = 0.065) D: low pre-test probability population per Sepsis-3 (pre-test=0.043).

Abbreviations: MDW, monocyte distribution width; WBC, white blood count


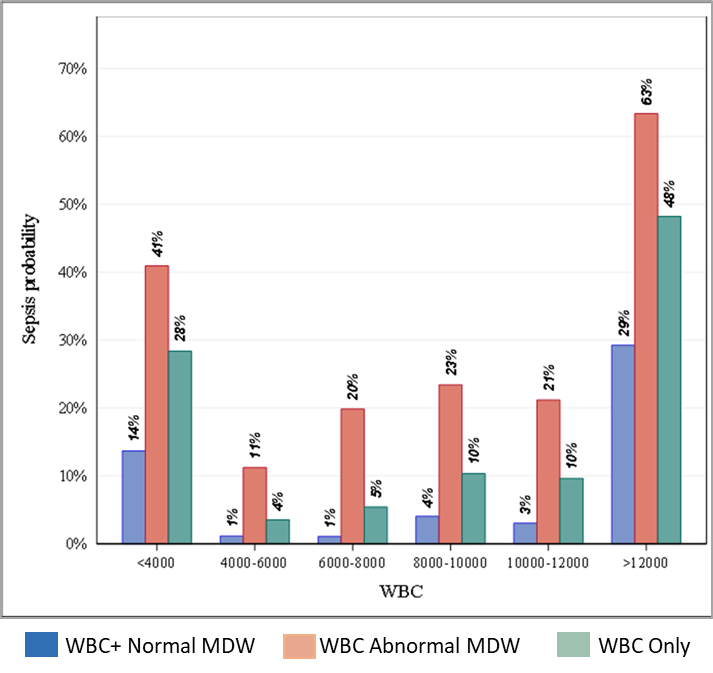


**A**


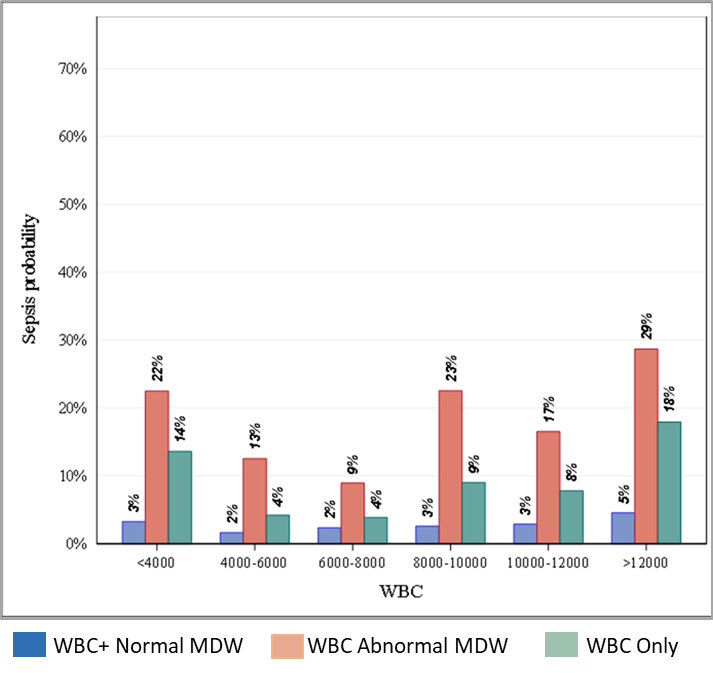


**B**

**C**


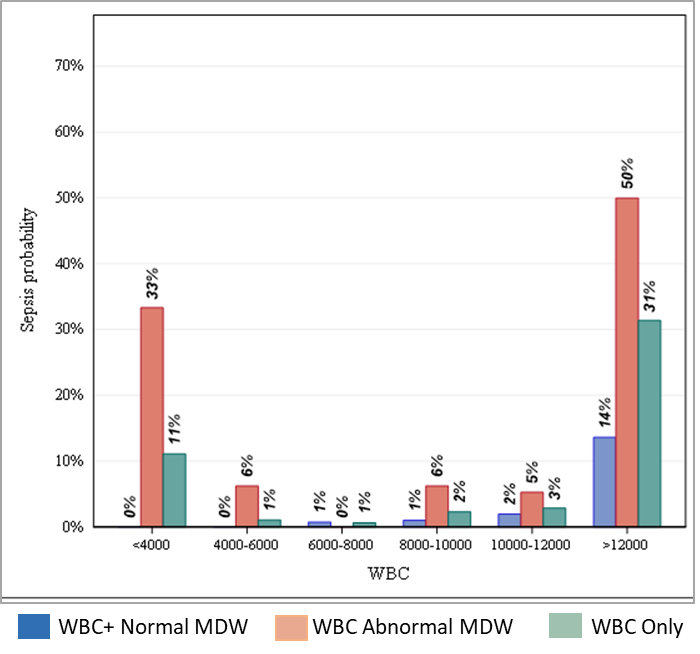


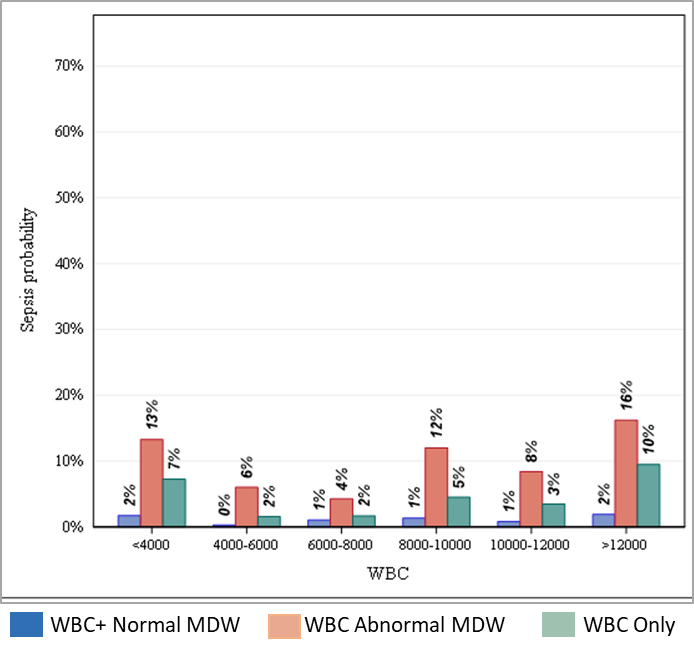


**D**
